# The NLRP3 Inflammasome: Role and Therapeutic Potential in Pain Treatment

**DOI:** 10.3389/fphys.2020.01016

**Published:** 2020-08-19

**Authors:** Hana Starobova, Evelyn Israel Nadar, Irina Vetter

**Affiliations:** ^1^Centre for Pain Research, Institute for Molecular Bioscience, University of Queensland, St Lucia, QLD, Australia; ^2^School of Pharmacy, The University of Queensland, St Lucia, QLD, Australia

**Keywords:** NOD, LRR and PYD domains-containing protein 3, neuro-inflammation, MCC950, interleukin-1β, sensory neurons, inflammatory diseases, interleukin-1 receptor

## Abstract

Pain is a fundamental feature of inflammation. The immune system plays a critical role in the activation of sensory neurons and there is increasing evidence of neuro-inflammatory mechanisms contributing to painful pathologies. The inflammasomes are signaling multiprotein complexes that are key components of the innate immune system. They are intimately involved in inflammatory responses and their activation leads to production of inflammatory cytokines that in turn can affect sensory neuron function. Accordingly, the contribution of inflammasome activation to pain signaling has attracted considerable attention in recent years. NLRP3 is the best characterized inflammasome and there is emerging evidence of its role in a variety of inflammatory pain conditions. In vitro and in vivo studies have reported the activation and upregulation of NLRP3 in painful conditions including gout and rheumatoid arthritis, while inhibition of NLRP3 function or expression can mediate analgesia. In this review, we discuss painful conditions in which NLRP3 inflammasome signaling has been pathophysiologically implicated, as well as NLRP3 inflammasome-mediated mechanisms and signaling pathways that may lead to the activation of sensory neurons.

## Introduction

Chronic pain is a dynamic process involving ongoing changes and adaption within the peripheral and central nervous systems and accompanies a vast number of inflammatory and non-inflammatory pathological states such as cancer, diabetes or rheumatoid arthritis.

Sensory neurons, including nociceptors, express a variety of ion channels and receptors involved in transformation of external stimuli into electrical signals, such the Transient Receptor Potential channels TRPV1,TRPV2, TRPV3, TRPA1, and TRPM8; purine receptor 3 (P2X_3_); voltage-gated sodium channel (Na_v_) subtypes Na_v_1.7, Na_v_1.8, and Na_v_1.9 and voltage-gated potassium channels (*K*_v_). The activation of these channels and receptors by thermal, mechanical or chemical nociceptive stimuli generate an action potential that is transmitted along the axons to the central nervous system. The immune system and neuro-inflammation play a crucial role in sensitization of the peripheral and central nervous systems, including activation of nociceptors. Specifically, microglia, satellite glia cells, Schwann cells, oligodendrocytes, keratinocytes, macrophages or T-cells releasing pro-inflammatory signaling molecules, such as interleukin-1β (IL-1β) or adenosine-triphosphate (ATP), are implicated in the pathology of various painful conditions (Ji et al., 2016; Inoue and Tsuda, 2018). Moreover, the depletion of macrophages reduces hyperalgesia in different rodent models of neuropathic pain (Barclay et al., 2007; Mert et al., 2009; Kobayashi et al., 2015; Zhang et al., 2016) and macrophages were shown to directly regulate the peristaltic activity of the colon via interaction with the enteric neurons and to release a variety of pain mediators such as nerve growth factor, tumor necrosis factor or IL-1β (Muller et al., 2014; McMahon et al., 2015).

Inflammasomes are a group of pattern recognition receptors (PRR) that detect pathogen- or damage-associated molecular patterns (PAMPs and DAMPs) and activate inflammatory responses. To date, there are six known inflammasome multi-protein complexes: NACHT, LRR and PYD/CARD domains-containing proteins - NLRP1, NLRP3, NLRP6, NLRP12, and NLRC4, and AIM2 (absent in melanoma 2) (Strowig et al., 2012). While the NLR family of the inflammasomes are fundamental component of the innate immune system, recognizing a broad spectrum of viral and bacterial stimuli, such as bacterial or viral RNA, lipopolysaccharides or uric acid crystals (Franchi et al., 2009b), AIM2 recognizes double stranded DNA released during various bacterial and viral infections (Sharma et al., 2019). Out of all the NLR inflammasomes, the NLRP3 inflammasome is amongst the best characterized, being predominantly expressed by immune cells such as macrophages and neutrophils and to a lesser extent by dendritic cells, microglia and dorsal root ganglia (Guarda et al., 2011b; Gustin et al., 2015; Fann et al., 2018). The activation of NLRP3 leads to release of IL-1β, a cytokine that is known to directly sensitize nociceptors and to cause pain (Binshtok et al., 2008). Increased NLRP3 expression and activation, together with the release of IL-1β, has been linked to pathogenesis of several painful inflammatory and non-inflammatory conditions such as rheumatoid arthritis, gout, neuropathic pain and diabetic wound healing (Table 1). In this review, we discuss the mechanisms leading to NLRP3 inflammasome activation in painful conditions, the resulting signaling pathways that can lead to the sensitization or activation of sensory neurons, as well as the therapeutic potential of NLRP3 inflammasome inhibition to mediate analgesia.

**TABLE 1:** NLRP3 in pain pathology.

**Disease / Condition**	**Description**	**Study description**	**Species**	**NLRP3 involvement**	**References**
Bladder pain syndrome/interstitial cystitis	Persistent pain or discomfort in bladder often caused by urinary tract infection.	Expression of Neurokinin-1 receptor and Substance P in nerve cells and bladder epithelial cells of the urinary bladder mucosa; examination of bladder pathology in knockout animals.	M (C57BL6/J, *Tlr4*^–/–^, *Il1b*^–/–^, *Asc*^–/–^, *Nlrp3*^–/–^)	Deteriorated bladder pathology in *Asc*^–/–^ and *Nlrp3*^–/–^ mice.	Butler et al., 2018
Burns pain	Pain caused by burn trauma associated with severe acute or chronic pain.	Assessment of burn-induced mechanical allodynia, thermal allodynia, edema and weight bearing in *Nlrp3^–/–^* and *Ice^–/–^* mice, and in C57BL6/J after administration of MCC950.	M (C57BL6/J, *Nlrp3^–/–^*, *Ice^–/–^*)	Attenuated weight bearing changes in *Nlrp3^–/–^* and mice treated with MCC950. No effect on mechanical or thermal allodynia.	Deuis et al., 2017
Metastatic cancer-induced bone pain (CIBP)	Bone pain caused by tumor metastasis associated with inflammation, hypercalcemia, skeletal fractures, compression of the spinal cord or nerves.	Assessment of CIBP induced mechanical allodynia (von Frey) and expression of NLRP3, ASC, Casp1 and IL-1β in spinal cord.	R	MCC950 treatment attenuated CIBP-related mechanical allodynia. MCC950 administration restored increased expression of NLRP3, ASC, Casp1 and IL-1β in spinal cord.	Chen et al., 2019
Chemo-therapy induced peripheral neuro-pathy	Neuropathy induced by damage of peripheral nerves by chemotherapy agents, manifested by pain, numbness, tingling, gait disturbances and loss of sensory discrimination.	Investigation of NLRP3 expression in spinal cord of oxaliplatin- and paclitaxel-induced peripheral neuropathy model.	M	No increased expression of NLRP3 after oxaliplatin or paclitaxel administration in spinal cord.	Tonkin et al., 2018
		Assessment of expression of NLRP3, Casp1 and IL-1β in DRG and SN. Assessment of mechanical allodynia after administration of phenyl-N-tert-butylnitrone (PNB) in paclitaxel-induced neuropathic pain model.	R	Increased expression of NLRP3, Casp1 and IL-1β in macrophages infiltrating in dorsal root ganglia and sciatic nerve. PNB attenuated mechanical allodynia and decreased NLRP3 expression in dorsal root ganglia and sciatic nerve.	Jia et al., 2017
		Investigation of paclitaxel effect on priming of the NLRP3 inflammasome.	M (C57BL6, *Nlrp3*^–/–^)	Paclitaxel primes the NLRP3 inflammasome via TLR4	Son et al., 2019
		Assessment of mechanical allodynia of mice in a bortezomid-induced peripheral neuropathy model and the intrathecal treatment with NLRP3 siRNA.	M	NLRP3 siRNA significantly prevented mechanical allodynia induced by bortezomib treatment.	Liu C. C. et al., 2018
		Assessment of the NLRP3 activation in oxaliplatin induced peripheral neuropathy model in rats treated with MCC950.	R	Increased activation of NLRP3 in astrocytes. MCC950 significantly reduces mechanical hyperalgesia following oxaliplatin treatment.	Wahlman et al., 2018
Cryopyrin associated periodic syndrome (CAPS)	Inherited autosomal dominant autoinflammatory disorder involving mutation in NLRP3; manifested by neurological symptoms such as headache and myalgia. Vast evidence of NLRP3 involvement in CAPS pathology, however no direct link of NLRP3 to pain pathology.	Case study of 13-year-old female with high fever, pericarditis, arthralgia, arthritis of the knees, abdominal pain and marked increase in inflammatory markers.	H	Mutation of the CIAS1/NLRP3 gene. No direct link of NLRP3 to pain symptoms.	Insalaco et al., 2014
		Forty-seven adult patients with CAPS with mutations in NLRP3 (CIAS1) gene and pathognomonic symptoms. Weekly subcutaneous injections of rilonacept (160 mg).	H	Rilonacept decreased the severity of key symptoms including joint pain and eye redness/pain.	Hoffman et al., 2008
		Case study of three family members with CAPS carrying mutation in NLRP3 (CIAS1) gene and treatment with anakinra.	H	Improved symptomatology after anakinra treatment including pain severity.	Maksimovic et al., 2008
		Case study of male patient with mutation of NLRP3 (CIAS1) gene treated with anakinra.	H	Improved pain scores and symptomatology following anakinra treatment.	Gerard et al., 2007
		Two siblings with mutation in NLRP3 (CIAS1) treated with anakinra.	H	Increased activity of Casp1 and levels of IL-1ß. Improved symptomatology following anakinra treatment.	Verma et al., 2010
Fibro-myalgia	Inherited autosomal dominant disease characterized by severe pain in the extremities.	Investigation of the role of coenzyme Q10 (CoQ10) and mitochondrial dysfunction in NLRP3 inflammasome activation in patients and rodent CoQ10 deficiency model.	H M	Increased expression of NLRP3 and IL-1ß in blood of patients with fibromyalgia that was reduced by treatment with CoQ10 treatment. Reduced NLRP3 inflammasome expression and activation in mice, accompanied with increased latencies in hot plate and tail flick test.	Cordero et al., 2014
Gout	Inflammatory arthropathy, associated with pain, caused by deposition of monosodium urate in the joints. Vast evidence of NLRP3 involvement in gout, however, no direct link of NLRP3 to pain pathology.	Investigation of uric acid crystals activation of NLRP3.	M (*Asc*^–/–^, *Casp1*^–/–^, *IL-1R*^–/–^)	Uric acid activates the NLRP3 and increases the production of IL-1β and IL-18. Deficient release of IL-1β in macrophages from *Asc*^–/–^ and *Casp1*^–/–^ mice following uric acid treatment.	Martinon et al., 2006
		Assessment of the effect of procyanidins on Monosodium Urate Crystals (MSU) treated Raw 264.7 cells and in rodent model of Gout in CD-1 mice.	CD-1 mice	Procyanidins attenuated gout pain and suppressed ankle swelling and inhibited MSU-induced activation of the NLRP3 inflammasome and increase of IL-1β.	Liu et al., 2017
		Ten patients with chronic active gouty arthritis treated with rilonacept.	H	Decrease of the pain score following rilonacept treatment.	Terkeltaub et al., 2009
Migraine	Chronic painful disorder characterized by attacks of severe headache and neurological symptoms.	Investigation of NLRP3 and IL-1ß expression and mechanical hyperalgesia in nitroglycerin induced chronic migraine model in mice treated with MCC950.	M	MCC950 reduced periorbital and hind paw mechanical hypersensitivity and restored increased expression of IL-1ß in trigeminal ganglia of nitroglycerin treated mice.	He et al., 2019
Neuro-pathic pain	Pain due to damage or disease affecting the peripheral or central nervous system; manifested as spontaneous pain (stimulus-independent pain) or pain hypersensitivity (stimulus-evoked pain).	Investigation of NLRP3 expression and mechanical hyperalgesia in spinal cord after chronic constriction injury (CCI) of the sciatic nerve and following the treatment with the adenosine triphosphate (ATP) release inhibitor Peptide5 (connexin-43 mimetic peptide).	M	Peptide5 restores the expression levels of NLRP3, ASC and cleaved CASP1 in spinal cord and attenuates mechanical allodynia following CCI of the sciatic nerve.	Tonkin et al., 2018
		Investigation of NLRP3, Casp1 and IL-1ß involvement and expression in spinal cord following partial sciatic nerve ligation (PSNL). Assessment of mechanical and thermal hyperalgesia.	M	PSNL induces overexpression of NLRP3, activation of Casp1 and release of IL-1ß in spinal cord.	Pan et al., 2018
		Investigation of the effect of MCC950 on heat and mechanical hyperalgesia in relapsing-remitting experimental encephalomyelitis (RR-EAE)-mouse model of MS-associated neuropathic pain.	M	MCC950 attenuates mechanical hyperalgesia of mice in RR-EAE-mouse model of MS-associated neuropathic pain.	Khan et al., 2018
		Investigation of the role of NLRP3 expression and IL-1β production in spared nerve injury (SNI).	M (C57BL6, *Nlrp3*^–/–^)	No increased expression of NLRP3 inflammasome components in the spinal cord at mRNA level. No difference in mechanical or thermal responses of C57BL6 or *Nlrp3*^–/–^ mice after SNI.	Curto-Reyes et al., 2015
Osteo-arthritis	Structural remodeling of the synovial joint manifested by pain, inflammation and disability.	Assessment of the effect of dexmedetomidine on NLRP3 expression, Casp1 activation and levels of IL-1ß and mechanical allodynia in model of osteoarthritis induced by papain.	M	Dexmedetomidine reduces knee inflammation, gait abnormalities, mechanical and heat hyperalgesia and the expression levels of NLRP3, activated Casp1 and IL-ß.	Cheng et al., 2019
Post-operative pain	Tissue trauma caused by surgery, involving peripheral and/or central nervous system, manifested by acute or chronic pain, neuropathic pain and hypersensitivity.	Investigation of NLRP3 inflammasome contribution to postoperative pain and assessment of mechanical sensitization.	M (C57BL6/J, *Nlrp3*^–/–^)	Only male, but not female, *Nlrp3*^–/–^ recover from surgery-induced mechanical sensitization faster than male and female C57BL6/J mice. Immune-mediated sex differences in postoperative pain.	Cowie et al., 2019
Rheuma-toid arthritis (RA)	Autoimmune chronic progressive disease resulting in deformity and pain of the joints of hands, feet, wrists and elbows. Vast evidence of NLRP3 involvement in rheumatoid arthritis pathology, however, no direct link of NLRP3 to pain pathology.	Investigation of NLRP3 activation in synovial tissues from RA and osteoarthritis patients and in rodent model of collagen-induced arthritis treated with MCC950.	M H	NLRP3 inflammasome pathway was activated in synovia of RA patients. MCC950 reduced joints inflammation, bone destruction, NLRP3 activation in the synovia and production of IL-1β.	Guo et al., 2018
		Investigation of A20 deficiency on NLRP3 activation in rodent RA model.	*M (A20^*myel–KO*^*, *Nlrp3*^–/–^, *Casp*^–/–^, *IL-1R*^–/–^)	A20 deficiency enhanced NLRP3 activation, pyroptosis and IL-1β secretion. Inflammation and cartilage destruction were reduced in *Nlrp3*^–/–^ and *Casp1*^–/–^ mice.	Vande Walle et al., 2014
		Assessment of expression levels of NLRP3, ASC, pro- and active Casp1, pro- and active IL-1β in blood of RA patients.	H	Increased expression of NLRP3, ASC, active – Casp1 and pro-IL-1β.	Choulaki et al., 2015
Schnitzler syndrome	Rare autoimmune disease that involves inflammation, fever, muscle, bone, and/or joint pain.	Six male patients diagnosed with Schnitzler syndrome and treated with anakinra.	H	Pain regression following anakinra treatment.	Szturz et al., 2014
Diabetes mellitus (DM) wound	Delayed wound healing, painful foot ulcers affecting quality of life in DM patients. Evidence of NLRP3 and IL-1β expression in DM wound pathology but no direct link to pain pathology.	Skin defects of 2 × 2 cm^2^ on the back of the rats. Quantification of ROS and NLRP3 in wound tissue.	R	Increased expression of NLRP3, IL-1β and ASC mRNA in wounds of diabetic rats.	Dai et al., 2017
		Assessment of mRNA and protein expression from chronic wound tissues from diabetic and non-diabetic patients of size >2 cm^2^ and <25 cm^2^	H DM patients Non DM subjects	Upregulation of NLRP3, caspase1 and IL-1β mRNA and protein levels in DM patient wounds.	Zhang et al., 2017

*R: rat, M: mouse, H: human, TLR4: toll-like receptor 4, IL-1ß: interleukin-1β, ASC: apoptosis speck-like protein, NLRP3: NOD, LRR, and PYD domains-containing protein 3, Casp1: caspase-1, DRG: dorsal root ganglia, SN: sciatic nerve, DM: diabetes mellitus.*

## Activation of the NLRP3 Inflammasome

**FIGURE 1:**
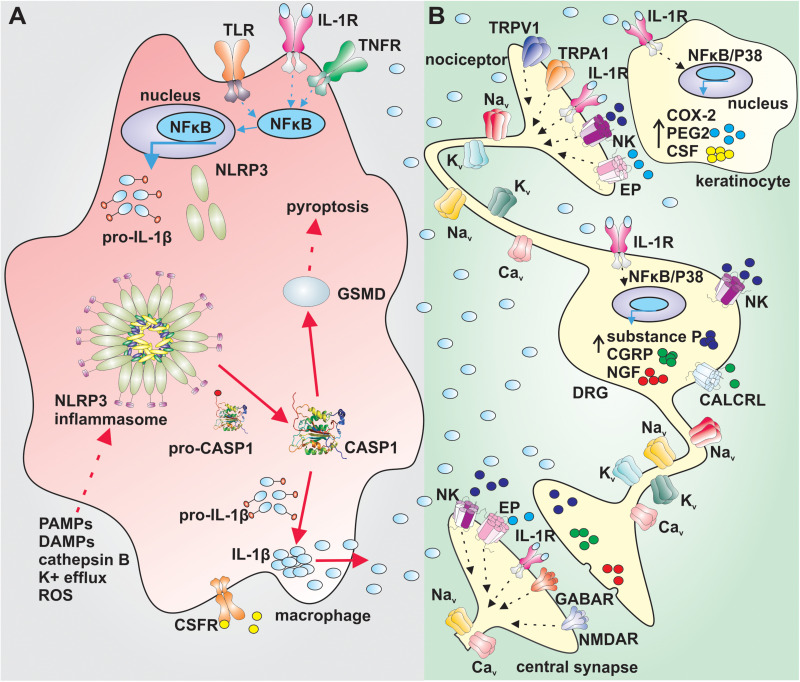
Putative mechanism of sensory nerve sensitization by IL-1β. **(A)** Priming of the NLRP3 inflammasome via activation of TLRs, IL-1R or TNFR in NFκB dependent manner in macrophages leads to increased expression of NLRP3 and pro-IL1β (blue arrows). The canonical activation of the NLRP3 (red arrows) by PAMPs, DAMPs, cathepsin B, K^+^ efflux or ROS leads to assembly of the NLRP3 inflammasome complex, activation of CASP1 and cleavage of pro-IL-1β and GSMD. Active IL-1β is released by macrophages and exerts its effect via IL-1R on sensory nerves and surrounding cells. Active GSMD initiates pyroptosis. **(B)** IL-1β increases the gene expression of COX-2, PEG2, and CSF in keratinocytes in a NFκB/P38 mitogen kinase dependent manner. PEG2 sensitize peripheral nerves and CSF regulates differentiation, proliferation and survival of macrophages. IL-1β increases excitability of nociceptors by altering the function of tetrodotoxin-resistant voltage-gated sodium channels via IL-1R and increases the gene expression in dorsal root ganglia in a NFκB/P38 mitogen kinase dependent manner. Substance P, CGRP, and NGF sensitize further peripheral nerves. IL-1β: interleukin 1β, TLR: toll-like receptor; IL-1R: interleukin-1 receptor; TNFR: tumor necrosis factor α receptor; NFκB: nuclear factor κB; NLRP3: NACHT, LRR, and PYD domains-containing protein 3; PAMPs: pathogen-associated molecular pattern; DAMPs: damage-associated molecular pattern; ROS: reactive oxygen species; CASP1: caspase 1; CSFR: colony-stimulating factor receptor; GSMD: gasdermin D; Na_v_: voltage gated sodium channel; EP: prostaglandin E receptor; NK: neurokinin receptor; CGRP: calcitonin gene-related peptide; CALCRL: calcitonin gene-related peptide receptor; NGF: nerve growth factor; COX-2: cyclooxygenase type 2; PEG2: prostaglandin E2; CSF: colony stimulating factor; NMDAR: N-methyl-D-aspartate receptor; K_v_: voltage gated potassium channel; Ca_v_: voltage gated calcium channel; TRPV1: transient receptor potential channel vaniloid; TRPA1: transient receptor potential channel ankyrin; GABAR: gamma-aminobutyric acid receptor.

The activation of the NLRP3 inflammasome requires, with some exceptions, two signals. The first signal leads to priming of the NLRP3 inflammasome by endogenous molecules (e.g., tumor necrosis factor (TNF) and IL-1β) or microbial components such as lipopolysaccharides. These compounds activate their cognate receptors, leading to nuclear factor-êB-dependent increase of NLRP3 and pro-IL-1β expression (Figure 1A) (Bauernfeind et al., 2009; Franchi et al., 2009a). The second signal causes the direct activation of NLRP3 and can involve several cellular signaling events such as potassium ion efflux, changes in calcium signaling, increase in production of reactive oxygen species by mitochondria or lysosomal leakage and release of cathepsin-B (Figure 1A; Brough et al., 2003; Bauernfeind et al., 2011; Munoz-Planillo et al., 2013; Orlowski et al., 2015). In the canonical NLRP3 signaling pathway, the activation of NLRP3 leads to assembly of a multi-protein complex that recruits caspase-1 via caspase activation and recruitment domain (CARD) interactions to promote caspase activation. Activated caspase-1 then cleaves pro-IL-1β and pro-interleukin-18 (pro-IL-18) into their active forms (Hu et al., 2013; Lu et al., 2014, 2015, 2016). Additionally, active caspase-1 cleaves the pore former pro-gasdermin D into its active form, inducing pyroptosis, a form of inflammatory cell death (Sagulenko et al., 2013).

The NLRP3 inflammasome can also be activated via non-canonical signaling pathways that were observed as a preferable response to gram-negative bacteria in mice, and are independent of toll-like receptor 4-dependent priming (TLR4) (Kayagaki et al., 2013). The non-canonical pathway involves the activation of caspase-11 by lipopolysaccharides, which leads to ATP efflux via pannexin-1 and subsequent activation of P2X-purinoceptor-7 (P2X7), efflux of intracellular potassium and activation of NLRP3. Caspase-11 also directly cleaves pro-gasdermin-D, inducing pyroptosis (Kayagaki et al., 2011; Shi et al., 2015; Yang et al., 2015).

Recently, another NLRP3 activation pathway, the so-called alternative inflammasome pathway, was discovered in human monocytes. This pathway is dependent on priming, independent of K^++^ efflux, involves caspase-8 activation and does not induce apoptosis-inducing speck-like protein containing CARD (ASC-speck) formation or pyroptosis (Gaidt et al., 2016).

## IL-1β-Induced Activation of Sensory Neurons

Activation of the NLRP3 inflammasome leads to release of active IL-1β, an inflammatory cytokine that regulates the function of various cells, such as immune and neuronal cells (Strowig et al., 2012; Figure 1B). IL-1β is released at the site of inflammation and is a well-known pain-inducing molecule. Several rodent studies have shown that intraplantar or intraperitoneal injection of IL-1β causes severe hyperalgesia (Ferreira et al., 1988; Amaya et al., 2006). IL-1β likely increases the excitability of nociceptors by altering the function of several neuronal ion channels and receptors, including transient receptor potential channels (TRPA1 and TRPV1), N-methyl-D-aspartate (NMDA) and gamma-aminobutyric acid (GABA) receptors, as well as voltage- gated K^+^, Na^+^, and Ca^2+^ channels (Binshtok et al., 2008; Schafers and Sorkin, 2008; Ren and Torres, 2009; Malsch et al., 2014; Stemkowski et al., 2015; Zanos et al., 2018). In this context, IL-1β can be considered to be a direct neuromodulator, and although the molecular signaling pathways leading to enhanced excitability have not been studied extensively, signaling via neuronal IL-1 receptors and activation of p38-mitogen-activated protein kinase appear to contribute mechanistically. Specifically, IL-1β alters the voltage-dependence of slow inactivation of tetrodotoxin (TTX)-resistant Na_v_ current in sensory neurons and also enhances persistent TTX-resistant current (Binshtok et al., 2008). Importantly, IL-1β-induced mechanical allodynia is also relieved in mice lacking the TTX-resistant isoform Na_v_1.9 (Amaya et al., 2006), supporting an important contribution of altered ion channel function to the neuromodulatory effects of IL-1β. Additionally, IL-1β also mediates production of endogenous molecules such as nerve growth factor, prostaglandin E2, cyclooxygenase-2 and calcitonin-gene related peptide that may further contribute to peripheral sensitization (Zucali et al., 1986; Safieh-Garabedian et al., 1995; Reinold et al., 2005; Figure 1B).

Increased levels of IL-1β accompany a vast number of inflammatory and non-inflammatory diseases and pathological states such as Alzheimer’s disease, cancer or rheumatoid arthritis (RA) (Mrak and Griffin, 2000; Lewis et al., 2006; Lo Gullo et al., 2014; Ruscitti et al., 2015). There is accumulating evidence for NLRP3 and cytokine expression in gout pathology as well, which is evidenced by studies conducted in animal models and human clinical studies (Martinon et al., 2006). A human monoclonal antibody targeting IL-1β that was used in randomized control trials showed significant reduction in risk of developing first gout flares as well as the prevention of recurring gout flares (Schlesinger et al., 2011). Several studies have shown increased expression of IL-1β in tissue and blood in rodent animal models of, for example, metastatic cancer-induced bone pain, chemo-therapy induced peripheral neuropathy, cryopyrin associated periodic syndrome, fibromyalgia, migraine and many other painful conditions (Verma et al., 2010; Cordero et al., 2014; Jia et al., 2017; Chen et al., 2019; He et al., 2019; Table 1). Additionally, IL-1β was found to be upregulated in the blood of patients suffering from painful conditions, and in tissues of rodents in various models of inflammatory or non-inflammatory pain (Ren and Torres, 2009; Gui et al., 2016), underpinning the importance of IL-1β in disease pathology. Only few studies used the global knockout of the IL-1β or IL1R, however, some of the studies did not assess pain behavior (Table 1). For example, a study investigating bladder pain syndrome in *Il1b^–/–^* mice showed no improvement in bladder pathology and pain (Butler et al., 2018) while studies in *IL-1R*^–/–^ mice assessing gout and rheumatoid arthritis did not investigate pathology or pain improvement (Martinon et al., 2006; Vande Walle et al., 2014). Moreover, increased levels of IL-1β do not necessarily involve activation of NLRP3 as IL-1β can also be released via alternative mechanisms, including activation of the NLRP1, NLRC4, and AIM2 inflammasomes (Martinon et al., 2002; Strowig et al., 2012).

Activation of NLRP3 also leads to release of active interleukin-18 (IL-18), which has been found to mediate painful conditions such as muscle pain, cancer-induced bone pain and neuropathic pain (Liu S. et al., 2018; Vasudeva et al., 2015; Yoshida et al., 2018). However, it is currently unclear whether IL-18 can also directly contribute to nociceptor sensitization, or whether these effects are secondary to other IL-18-induced signaling events.

## NLRP3 in Pain Pathology

The NLRP3 inflammasome has been implicated in the pathology of many painful diseases and conditions such as bladder pain, neuropathic pain, rheumatoid arthritis, gout, migraine and many more (Table 1). With increasing insight into the role of the immune system in the pathology of multiple diseases, it is perhaps not surprising that NLRP3 has also been suggested as a driving factor in the development of many painful diseases. However, despite the clear links between inflammation and pain, as well as NLRP3 and inflammation, relatively few studies have directly assessed the contribution of NLRP3 signaling to pain.

A prototypical NLRP3-linked condition is cryopyrin-associated periodic syndrome (CAPS), an inherited autosomal dominant autoinflammatory disorder characterized by recurrent episodes of inflammation and fever. CAPS patients carry mutations in the NLRP3 (CIAS1) gene leading to activation of the NLRP3 inflammasome and overproduction of IL-1β. Accordingly, treatment with rilonacept, a soluble IL-1 decoy receptor, or anakinra, an IL-1 receptor antagonist, improve the symptomatology of those patients. However, there is only limited – though promising – evidence regarding efficacy of these treatments against painful symptoms often reported by CAPS patients, such as headaches and myalgia (Gerard et al., 2007; Hoffman et al., 2008; Maksimovic et al., 2008; Verma et al., 2010).

Increased expression of NLRP3 and IL-1β was also found in blood of patients suffering from fibromyalgia, and treatment with coenzyme Q10 decreased both overexpression of NLRP3 as well serum IL-1β and IL-18 levels (Cordero et al., 2014). Similar mechanisms were also observed in a rodent model of fibromyalgia, where coenzyme Q10 decreased pain behaviors in the hot plate and tail flick tests (Cordero et al., 2014).

In common inflammatory disorders characterized by pain as a predominant symptom, such as rheumatoid arthritis, osteoarthritis and gout, release of cytokines including IL-1β is well-known to contribute to pathology. Accordingly, treatments aimed at inhibiting IL-1β effects – including with biologics such as anakinra, canakinumab and rilanocept – is a common therapeutic approach that shows some promise in alleviating painful symptoms (Martinon et al., 2006; Terkeltaub et al., 2009; Vande Walle et al., 2014; Choulaki et al., 2015; Liu et al., 2017; Guo et al., 2018; Cheng et al., 2019). However, these studies provide only limited evidence about the analgesic effect of these substances. Similarly, Schnitzler syndrome, a rare autoimmune disease characterized by joint pain and arthritis, was linked to rare NLRP3 gene mutations and increased IL-1β release, and treatment with anakinra caused pain regression in six male patients (Szturz et al., 2014; Pathak et al., 2019). However, while NLRP3 has been implicated in the pathology of these conditions, IL-1β can also be released upon activation of NLRP1, NLRC4, and AIM2 inflammasomes (Martinon et al., 2002; Terkeltaub et al., 2009; Strowig et al., 2012; Liu et al., 2017; Cheng et al., 2019). Thus, the direct contribution of NLRP3 to IL-1β release, and pain, in these conditions remains to be unequivocally demonstrated. Illustrating this point is the observation that burns pain – which is accompanied by severe inflammation, including increased levels of IL-1β (Ono et al., 1995; Vindenes et al., 1998) – developed normally in animals lacking NLRP3 (*Nlrp3*^–/–^) or caspase-1/caspase11 (*Ice*^–/–^) (Deuis et al., 2017).

Varied outcomes were also reported in studies assessing the contribution of NLRP3 to neuropathic pain, which may be accompanied by significant neuro-inflammation. Increased expression and activation of NLRP3 as well as increased production of IL-1β were reported in several rodent models of nerve injury and chemotherapy-induced neuropathic pain (Jia et al., 2017; Khan et al., 2018; Liu C. C. et al., 2018; Pan et al., 2018; Tonkin et al., 2018; Wahlman et al., 2018; Son et al., 2019). However, while NLRP3 expression and activation was increased after chronic constriction injury of the sciatic nerve and partial sciatic nerve ligation, spared nerve injury did not result in elevated expression of NLRP3 inflammasome components, and pain behaviors of *Nlrp3*^–/–^ mice were unaltered (Curto-Reyes et al., 2015; Pan et al., 2018; Tonkin et al., 2018). Similarly, investigations of the expression and activation of NLRP3 in models of oxaliplatin-induced neuropathy resulted in seemingly contradictory findings (Tonkin et al., 2018; Wahlman et al., 2018). These studies highlight the need for careful consideration of experimental procedures – including the type of injury, cell types included in analysis and methods used to assess expression and activity of NLRP3 – in preclinical studies addressing the contributions of the NLRP3 inflammasome to painful conditions.

While publications claiming direct or indirect NLRP3-modulating effects by analgesic compounds are plentiful, relatively few studies have assessed the contribution of NLRP3 signaling to pain using highly selective NLRP3 modulators or *Nlrp3*^–/–^ animals. These approaches have revealed a key contribution of NLRP3 to migraine, with MCC950, a specific NLRP3 antagonist, reducing periorbital and hind paw mechanical hypersensitivity and reversing increased expression of IL-1β in trigeminal ganglia (He et al., 2019). Similarly, treatment with MCC950 attenuated mechanical allodynia in a rodent model of cancer-induced bone pain, and in oxaliplatin-induced peripheral neuropathy model and restored the increased expression levels of NLRP3, ASC, caspase-1 and IL-1β in spinal cord to basal levels (Wahlman et al., 2018; Chen et al., 2019). Interestingly, Cowie et al. showed that the deletion of the NLRP3 gene causes immune-mediated sex differences in pain behavior, as male *Nlrp3*^–/–^ mice recovered from surgery-induced behavioral and mechanical sensitization faster than female *Nlrp3*^–/–^ mice (Cowie et al., 2019).

## Therapeutic Potential of NLRP3 Modulators for Pain Treatment

The complex signaling cascades associated with NLRP3 activation lend themselves to pharmacological intervention at multiple points, including direct inhibition of NLRP3 activation, inhibition of inflammasome assembly, inhibition of caspase-1 or gasdermin-D, or inhibition of the biological effects of downstream effectors such as IL-1β. For the latter, a number of approved modulators – including IL-1 receptor antagonists or inhibitory antibodies – are clinically available, as outlined above. However, as IL-1β production is the result of several convergent signaling pathways, inhibition of the biological activities of IL-1β may result in unintended immunosuppressive effects. Accordingly, a number of specific NLRP3 inhibitors with various mechanisms of NLRP3 inhibition have been reported, some of which are currently in clinical development [for review see Zahid et al. (2019); Jiang et al. (2020); Olcum et al. (2020); Zhang et al. (2020))].

The small molecule MCC950 inhibits the canonical and non-canonical activation of the NLRP3 inflammasome (Coll et al., 2015) while the ketone metabolite β-hydroxybutyrate inhibits only canonical activation of the NLRP3 inflammasome via inhibition of potassium efflux (Youm et al., 2015). The type I interferons, including IFN-α and IFN-β, inhibit the NLRP3-dependent production of IL-1β and IL-18 through a yet unknown mechanism (Guarda et al., 2011a) and IFN-β therapy of multiple sclerosis in patients reduces IL-1β levels and NLRP3 protein expression (Malhotra et al., 2015). Additionally, the induction of autophagy by resveratrol, or the inhibition of NLRP3 expression by microRNA-223, inhibits NLRP3 activation and the production of IL-1β (Bauernfeind et al., 2012; Chang et al., 2015).

However, despite overwhelming evidence linking the NLRP3 inflammasome to IL-1β and IL-18 production and inflammation, and convincing evidence that these cytokines can in turn mediate pain, the therapeutic potential of compounds targeting NLRP3 inflammasome signaling pathways remain to be assessed. Certainly, for some painful conditions such as cancer-induced bone pain, chemotherapy- or multiple sclerosis-induced neuropathy, migraine and fibromyalgia, inhibition of NLRP3 signaling appears to be a promising pain treatment strategy. However, only few clinical studies using selective NLRP3 inhibitors are in progress, and additional preclinical studies are required to increase our understanding of the NLRP3-dependent mechanisms contributing to peripheral or central sensitization and pain. Additionally, as activation of the NLRP3 inflammasome regulates immune responses, it is important to consider putative side effect that could arise from NLRP3 inhibition. For example, NLRP3 activation is a first defensive response to bacterial infections, suggesting that NLRP3 inhibitors could be associated with an increased susceptibility to infections. Moreover, inhibition of NLRP3-dependent IL-1β release may affect tumor progression. For example, tumor progression and metastasis of melanoma, gastric and colon cancer were shown to be driven by IL-1β (Krelin et al., 2007; Okamoto et al., 2010; Dunn et al., 2012; Li et al., 2012), whereas the inhibition of IL-1β delayed the regression of the murine B16 melanoma hepatic metastases (Vidal-Vanaclocha et al., 1994). Moreover, the treatment of patients or mice, with metastatic breast cancer, with anakinra, a specific IL-1R inhibitor, significantly reduced the tumor growth (Holen et al., 2016; Wu et al., 2018). Therefore, use of NLRP3 or IL-1β inhibitors for cancer- or chemotherapy-associated pain requires careful investigation and consideration.

In summary, our understanding of the therapeutic potential of NLRP3 inhibition for the treatment of various painful disorders remains limited, despite emerging evidence that these targeted anti-inflammatory approaches may provide benefit. It is likely that analgesic effects will be realized for selected pain types, or subgroups of patients. Undoubtedly, with an increasing number of NLRP3 inhibitors entering clinical trials, significant insights into the therapeutic potential of these treatment approaches will be gained from additional pre-clinical and clinical studies.

## Conclusion

NLRP3 inflammasome activation is increasingly recognized as a key molecular event underpinning numerous inflammatory conditions, including those associated with significant pain. However, our understanding of molecular mechanisms leading to activation of the NLRP3 inflammasome, and subsequent effects on sensory systems, remains limited. Accordingly, the involvement of NLRP3 in the development of specific painful conditions, and the positive as well as negative effects of NLRP3 inhibition on human health remain to be determined in future studies.

## Author Contributions

HS and EN wrote the manuscript. IV edited the manuscript. HS designed figures and tables. All authors contributed to the article and approved the submitted version.

## Conflict of Interest

The authors declare that the research was conducted in the absence of any commercial or financial relationships that could be construed as a potential conflict of interest.
